# Effect of L-Carnitine Supplementation on *Apelin* and *Apelin Receptor (Apj)* Expression in Cardiac Muscle of Obese Diabetic Rats

**DOI:** 10.22074/cellj.2018.5408

**Published:** 2018-05-28

**Authors:** Neda Ranjbar Kohan, Saeed Nazifi, Mohammad Reza Tabandeh, Maryam Ansari Lari

**Affiliations:** 1 Department of Clinical Studies, School of Veterinary Medicine, Shiraz University, Shiraz, Iran; 2 Department of Biochemistry and Molecular Biology, Faculty of Veterinary Medicine, Shahid Chamran University of Ahvaz, Ahvaz, Iran; 3 Stem Cells and Transgenic Technology Research Center, Shahid Chamran University of Ahvaz, Ahvaz, Iran; 4 Department of Food Hygiene, School of Veterinary Medicine, Shiraz University, Shiraz, Iran

**Keywords:** *Apelin*, *Apelin Receptor*, Cardiac Muscle, Diabetes, L-Carnitine

## Abstract

**Objective:**

L-carnitine (LC) has been shown to protect cardiac metabolism in diabetes patients with cardiovascular diseases (CVDs). Apelin, a newly discovered adipocytokines, is an important regulator of cardiac muscle function; however, the role of the level of expression of Apelin axis in improvement of cardiac function by LC in diabetic patients, is not clear. In the present study, obese insulin-resistant rats were used to determine the effect of LC, when given orally with a high-calorie diet, on *Apelin* and *Apelin receptor (Apj)* expression in cardiac muscle.

**Materials and Methods:**

In this experimental study, rats were fed with high-fat/high-carbohydrate diet for five weeks and subsequently were injected with streptozotocin 30 mg/kg for induction of obesity and insulin resistance. After confirming the induction of diabetes (serum glucose above 7.5 mmol/L), the animals received LC 300 mg/kg in drinking water for 28 days. On days 0, 14 and 28 after treatment, cardiac *Apelin* and *Apj* gene expression was evaluated by real time polymerase chain reaction (PCR) analysis. Serum levels of insulin, Apelin, glucose, tumor necrosis factor-α (TNF-α), interleukin-1β (IL-1β) and the homeostasis model assessment of insulin resistance (HOMA-IR) were also measured using commercial kits.

**Results:**

Cardiac *Apelin* and *Apj* expression and serum Apelin were increased in obese rats, while LC supplementation decreased the serum levels of *Apelin* and down-regulated *Apelin* and *Apj* expression in cardiac muscle. These changes were associated with reduced insulin resistance markers and serum inflammatory factors and improved lipid profile.

**Conclusion:**

We concluded that LC supplementation could attenuate the over-expression of Apelin axis in heart of diabetic rats, a novel mechanism by which LC improves cardiovascular complications in diabetic patients.

## Introduction

Obesity is associated with a variety of inflammatory- related diseases, such as insulin resistance (IR), type 2 diabetes and cardiovascular diseases (CVD) (1). Risk of coronary heart disease (CHD) and stroke is reported to be higher in obese subjects in comparison to normal weight people (2). The pathogenesis of CVD in obese patient is very complex; however, adipose tissue dysfunction is considered to be the central mechanism involved in the development of CVD including atherosclerosis and cardiomyopathy (3, 4).

Adipocytokines or adipokines are adipose tissue-derived hormones that act as pro-inflammatory, vasoactive, and cytokine-like hormones (5-8). It has been shown that these immunomodulatory proteins act as modulators of metabolic and cardiovascular processes (5, 7). Based on both animal and human studies, it has been reported that dysregulation of adipocytokine secretion caused by excess adiposity and dysfunctional adipocytes, can play a pivotal role in obesity-related CVDs. Though adipocytokines are mainly secreted by adipose tissue, they are also expressed and secreted by various cardiovascular tissues such as cardiomyocytes and endothelial cells and regulate cardiacovascular *function* via a distinct *paracrine* mechanism (3, 4). 

Apelin is a novel adipokine which is produced from a 77-amino acid precursor. Different active forms of Apelin including Apelin-12, Apelin-13, Apelin-17, Apelin-19 and Apelin-36 have been reported. In different tissues, Apelin-36 is the most widely expressed form, while Apelin-13 is more potent and more abundant in the circulation (8, 9). It is the endogenous ligand of the orphan receptor angiotensin like-receptor 1 (AGTRL1), a G-protein-coupled receptor that has been found to be involved in various physiologic events, such as insulin sensitivity, glucose homeostasis and regulation of the cardiovascular function (10, 11). Apelin is upregulated by insulin and inhibits pancreatic insulin secretion (9, 12-14). In clinical and experimental studies, serum levels of Apelin or its adipose tissue expression are increased in case of obesity and insulin resistance (5, 15, 16). It is also involved in inflammatory responses in obese subjects and its expression is positively associated with some inflammatory markers such as tumor necrosis factor-α (TNF-α), interleukin-1ß (IL-1ß) (17, 18). 

Recent findings have shown the role of Apelin in cardiovascular functions. A high level of Apelin expression has been reported in cardiac muscles of rats and humans (19). Apelin stimulates inotropic potential of cardiac muscle cells and increases coronary blood flow by vascular dilation (20). Protective effect of Apelin has been reported against age-related progressive cardiac dysfunction in Apelin-deficient mice (21). Moreover, Apelin expression increases in the arteries of patients with atherosclerosis and chronic heart failure (22, 23). 

Although application of lipotropic agents for prevention of cardiovascular disease has been confirmed in previous research, data about their effects on adipokine expression in cardiovascular system is limited (5). L-carnitine (L-bhydroxy- 4-N-trimethylaminobutyric acid) (LC) is an amino acid derivative that plays an important role in energy production in the myocardium and is considered an essential cofactor for fatty acid ß-oxidation in the heart (24, 25). It has been found that LC has favorable effects in patients with severe insulin resistance and cardiovascular disorders, such as CHD, chronic heart failure and peripheral vascular disease. In patients with ischemic heart disease, LC reduces the myocardial injury mainly through improving carbohydrate metabolism and reducing the toxicity of high levels of free fatty acid (25, 26). 

Currently, it is not clear that LC improves obesity- associated cardiovascular complications through local alteration of Apelin system in myocardial tissue, or via an endocrine adaptation that is reflected by a change in serum levels of Apelin. The aim of the present study was to evaluate the gene expressions of *Apelin* and *Apelin receptor* in cardiac muscle of high-fat diet treated diabetic rats and their association with inflammatory and insulin resistance markers. 

## Materials and Methods

To perform this experimental study, 60 male Wistar rats (200 ±12 g) were obtained from the center of laboratory animals of the Faculty of Veterinary Medicine of Shahid Chamran University, Ahvaz, Iran. They were housed in a temperature-controlled room (at 23 ± 1°C) with 12 hour light/dark cycles and they had free access to rat chow (Pars, Iran) and water at libitum. The rats experienced 7 days of acclimatization before initiation of the experiment.

This experiment was accomplished under the approval of the State Committee on Animal Ethics, Shiraz University, Shiraz, Iran. The recommendations of European Council Directive (86/609/EC) of November 24, 1986, regarding protection of animals used for experimental purposes, were also followed. 

### Experimental design

Animals (n=60) were randomly divided into four groups (n=15). Two groups were fed with high-energy diet [prepared by adding 20% sucrose (w/w) and 10% beef tallow (w/w) into diets] for 5 weeks and called as High fat/High carbohydrate (HF/HC) (n=30), whereas the other ones consumed normal diets for the same period and served as control groups (n=30). After 5-week administration of HF/HC diets, animals were treated with a single injection of streptozotocin (STZ, Sigma, Germany) 30 mg/kg body weight. Five days after STZ treatment, glucose was measured by a glucometer (EasyGluco, South Korea) and diabetes induction was confirmed if serum glucose was above 7.5 mmol/l. The day after diabetes confirmation, was considered day 0 of LC treatment. One diabetic group was treated with 300 mg/kg/day LC (n=15) in drinking water concomitant with HF/HC diets for 28 days, while the other diabetic group (n=15) (diabetic control) was fed only with HF/HC diets for the same period. One control group (n=15) received normal diet and the other control group (n=15) (LC-treated control) consumed 300 mg/kg LC in drinking water for 28 days. 

### Sampling 

Serum and tissues were taken on days 0, 14 and 28 after diabetes induction and LC treatment. Animals were euthanized with a combination of 100 mg/kg of ketamine and 10 mg/kg of xylazine. Blood samples were collected immediately, and sera were separated and stored at -20°C until used. Cardiac muscles were separated, surrounding tissues were removed and kept at -70°C until used. Absolute body weight of each rat from each group was measured at the end of the HF/HC feeding and LC treatment period.

### Plasma biochemical assays

The plasma glucose, triglyceride (TG), cholesterol, high density lipoprotein-cholesterol (HDL-C) and low density lipoprotein-cholesterol (LDL-C) levels were determined using commercially available kits (Pishtazteb, Iran). Serum levels of Apelin (EastBiopharm, Mainland, China) and insulin (KOMA BIOTECH INC, South Korea) were measured by rat specific ELISA kits using a multiplate ELISA reader (BioTek, CA, USA). The sensitivity of the assays for insulin was 0.75 µIU/ml. TNF-α and IL1ß levels in the serum were determined using ELISA kits specific for rat (KOMA BIOTECH INC, South Korea). The sensitivities of the assays for TNF-α and IL-1ß were 45 pg/ml and 15 pg/ml, respectively.

### Insulin resistance estimation

The homeostasis model assessment of basal insulin resistance (HOMA-IR) was used to calculate an index from the product of the fasting concentrations of plasma glucose (mmol/l) and plasma insulin (µU/ ml) divided by 22.5 (23). Lower HOMA-IR values indicated greater insulin sensitivity, whereas higher HOMA-IR values indicated lower insulin sensitivity (i.e. insulin resistance) (26).

### Isolation of total RNA and synthesis of cDNA 

Total RNA was isolated from 100 mg of cardiac muscles using RNX ^TM^ isolation reagent according to the manufacturer’s procedure (CinaClon, Iran). Possible DNA contamination was removed by treatment of RNA (1 µg) with DNase I (2 U/µl) for 1 hour at 37oC (Vivantis, Malaysia). Concentration of extracted RNA was calculated at the wavelength of 260 nm using NanoDrop spectrophotometer (Eppendorf, Germany). To detect the purity of RNA, its optical density (OD) ratio at 260/280 nm was determined and samples having a ratio >1.8 were used for cDNA synthesis. Reverse transcription was carried out using the RocketScript RT PreMix kit using 1 µg of RNA and random hexamer primers based on manufacturer’s protocol (Bioneer Corporation, South Korea). Reverse transcription was carried out at 42°C for 90 minutes followed by incubation at 70°C for 5 minutes. cDNAs were stored at -20°C until used for real-time polymerase chain reaction (PCR). 

### Real time polymerase chain reaction analysis

Relative quantitative analysis of target genes (*Apj* and *Apelin*) and an internal reference gene (Gapdh) was done using the realtime PCR system (Light-Cycler 480, Roche, Germany). Specific sets of primers (Bioneer, South Korea) designed for this study were: 

Apelin (GenBank accession NO: NM_031612.3):

F: 5'-TGGAAGGGAGTACAGGGATG-3'

R: 5'-TCCTTATGCCCACT-3'

Apj (GenBank accession NO: NM_031349.2):

F: 5'-GGACTCCGAATTCCCTTCTC-3'

R: 5'-CTTGTGCAAGGTCAACCTCA-3'

Gapdh (GenBank accession NO: NM_NM-001034055): 

F: 5'-CTCATCTACCTCTCCATCGTCTG-3'

R: 5'-CCTGCTCTTGTCTGCCGGTGCTTG-3'.

Final reaction volume for the analysis of *Apelin* and *Apj* gene expression was 12.5 µL (containing 6.25 µl qPCR^TM^ Green Master Kit for SYBR Green I^®^ (Jena Biosciense, Germany), 0.25 µl of each primer (200 nM), 3 µl cDNA (~100 ng), and 2.25 µl nuclease-free water). The cycling conditions were 95°C for 5 minutes, followed by 45 cycles at 95°C for 15 seconds and 60°C for 30 seconds. Reactions were performed in triplicate. All runs included one negative-template control consisting of PCR-grade water instead of cDNA. Relative quantification was performed according to the comparative 2^-ΔΔCt^ method and using Lightcycler 96^®^ software. Validation of assay to ensure that the primer used for the target and internal reference genes had similar amplification efficiencies, was performed. All qPCR analysis was performed according to The Minimum Information for Publication of Quantitative Real-Time PCR Experiments (MIQE) guideline (27).

### Cell lysis and Western blot analysis

The levels of Apelin and Apj proteins in the heart of treated and untreated rats on day 28 were determined using Western blot analysis. Briefly, 50 mg of tissues were incubated for 30 minutes at 4°C in 1 ml homogenization buffer (pH=7.4) containing 255 mM sucrose, 2 mM EDTA and 20 mM HEPES supplemented with protease inhibitor cocktail (Roche, Laval, Canada) and homogenized with homogenizer (Silent Crusher, Heidolph, Germany) on ice. Homogenates were centrifuged at 12000 g for 15 minutes at 4°C, supernatants were collected and protein contents were determined using Bradford assay kit (Pars Azma, Iran). Next, 25 µl of cell lysate was mixed with 25 µl sodium dodecyl sulphate (SDS) sample loading buffer (0.5 M Tris, pH=6.8, 50% glycerol, 10% SDS, 7.5% 2-ß mercaptoethanol, and 0.2% bromophenol blue). The final concentration of protein in each sample was about 5 µg/µl. The samples were boiled for 10 minutes at 65°C, loaded on 10% SDS-polyacryleamide gel electrophoresis (SDS-PAGE) and electrotransferred onto a nitrocellulose membrane. (Schleicher & Schuell, Inc., Keene, NH). The membranes were blocked (for 1 hour) in Tris buffered saline (TBS) containing 0.05% Tween 20 (TBST, pH=7.4) and nonfat dry milk (5%). Blots were then washed in TBS and incubated with primary antibodies (anti Apelin, anti APJ, and anti GAPDH, Abcam, Cambridge, UK) at 1:200 dilution. Primary antibodies were detected by using goat anti-rabbit horseradish peroxidase conjugated antibody (Abcam, Cambridge, UK, 1:1000 dilution) and DAB reagent (Sigma Aldrich, Germany).

### Statistical analysis

Statistical analysis was conducted using SPSS 18 software. Descriptive statistics were presented as means ± SE. Means of each variable in the treatment groups and at various time points were compared using two-way analysis of variance. Group, time and their interaction term were considered as fixed effects in the model. In significant cases, adjusted comparison of means was undertaken using Sidak post-hoc test. In case of high variability among data and non-homogenous variance, transformation of data was performed. For most factors, variance became homogenous after logarithmic transformation. However, comparison of weight of animals among experimental groups on each day, was performed using non-parametric analysis of variance (Kruskal-Wallis test) followed by Mann-Whitney U test. In all analysis, a P<0.05 was considered statistically significant.

## Results

### L-carnitine supplementation and insulin resistance markers

**Fig.1:**
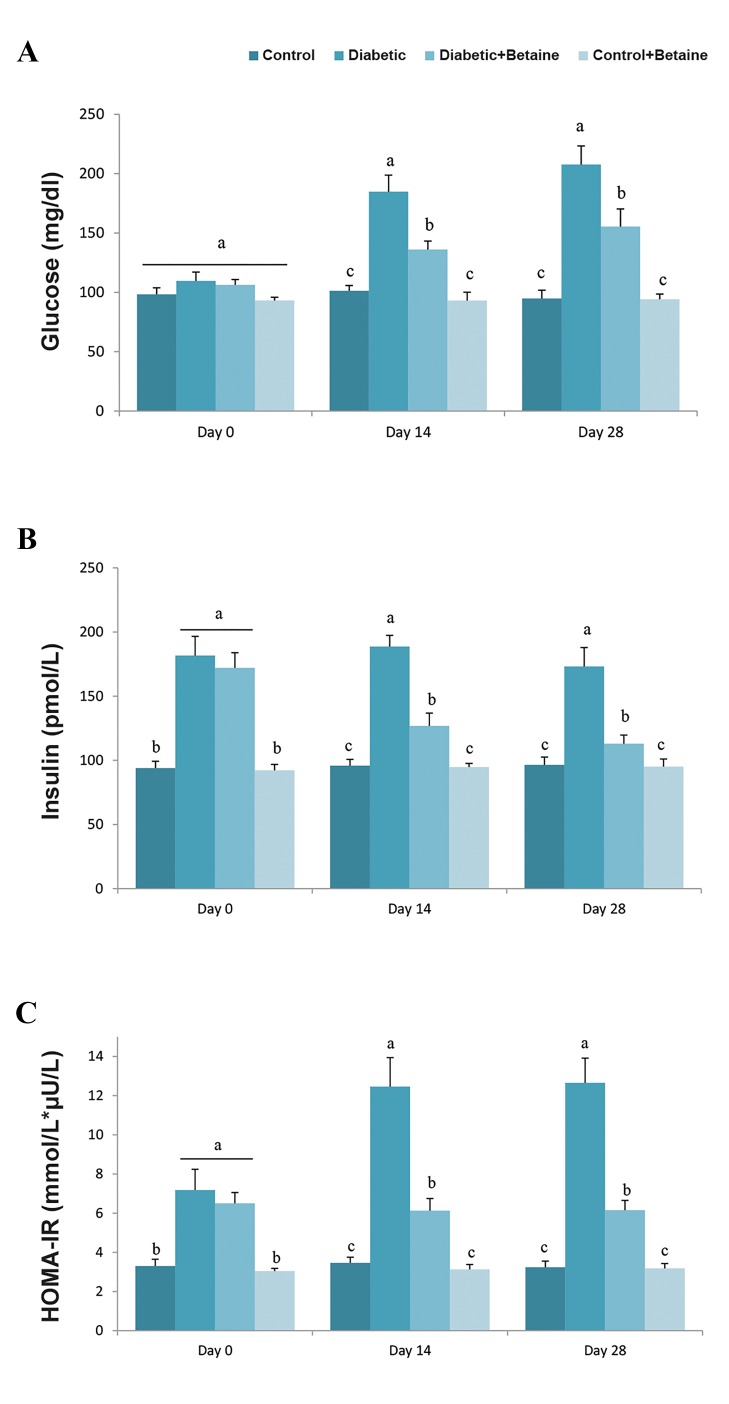
Effect of LC on glucose, insulin and HOMA-IR levels in HF/HC diet fed diabetic rats. Rats were fed with regular chow diet (control) or HF/ HC diet for 5 weeks (diabetic). HF/HC fed rats were treated with LC (300 mg/kg/day) from the first day of diabetes confirmation for 14 or 28 days (Diabetic+LC, n=5/group). A. Serum glucose levels, B. Serum insulin level, and C. HOMA-IR levels. Data are expressed as means ± SE. Different letters (a, b and c) demonstrate significant differences between groups on each day at P<0.05. LC; L-carnitine, HOMA-IR; Homeostatic model assessment of insulin resistance, and HF/HC; High fat/high carbohydrate.

Serum glucose level showed no significant changes after 5weeks of feeding with HF/HC, while it significantly increasedafter STZ treatment (Fig .1A). Higher levels of insulin andHOMA-IR were found in diabetic group after diabetesinduction as compared to control group (P<0.05, Fig .1B, C). These results showed that HF/HC diet and STZ treatment ledto obvious insulin resistance with higher insulin, glucose andHOMA-IR levels compared to control animals. Treatmentof diabetic rats with LC for 14 or 28 days could improvehyperglycemia, hyperinsulinemia and elevated HOMA-IR, in particular, 28 days after LC supplementation (Fig .1A-C).

### L-carnitine supplementation and body weight change

**Fig.2:**
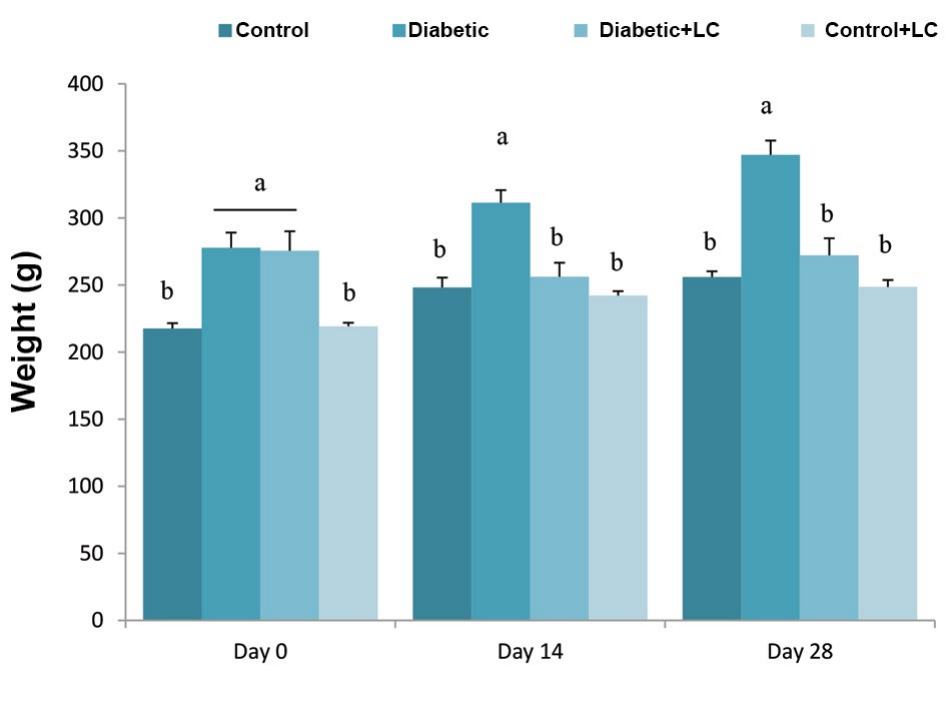
Effect of LC on body weight change in HF/HC diet fed diabetic rats. Rats were fed with regular chow diet (control) or HF/HC diet for 5 weeks (diabetic). HF/HC fed rats were treated with LC (300 mg/kg/day) from the first day of diabetes confirmation for 14 or 28 days (Diabetic+LC, n=5/ group). Data are presented as means ± SE. Different letters (a, b and c) demonstrate significant differences among groups on each day at P<0.05. LC; L-carnitine and HF/HC; High fat/high carbohydrate.

Significant difference was observed in body weight between the HF/HC-fed group and the group fed with regular diet. Feeding of rats with high calorie diet for five weeks resulted in elevation of body weight compared to the control group in a time-dependent manner (P<0.05, Fig .2). Diabetic rats treated with LC for 14 or 28 days showed no significant changes in body weight (P>0.05, Fig .2). Also, 28-day treatment with LC did not change the body weight of healthy rats. 

### L-carnitine supplementation and alteration of serum levels of Apelin

**Fig.3:**
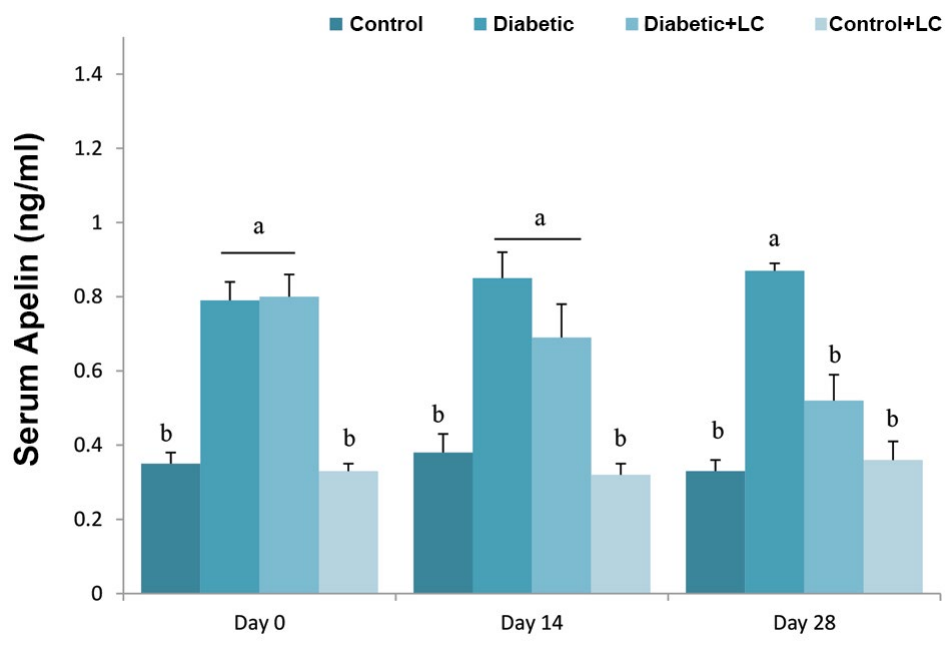
Effect of LC on serum Apelin level in HF/HC diet fed diabetic rats. Rats were fed with regular chow diet (control) or HF/HC diet for 5 weeks (diabetic). HF/HC fed rats were treated with LC (300 mg/kg/day) from the first day of diabetes confirmation for 14 or 28 days (Diabetic+LC, n=5/ group). Data are expressed as means ± SE. Different letters (a, b and c) demonstrate significant differences among groups on each day at P<0.05. LC; L-carnitine and HF/HC; High fat/high carbohydrate.

Compared to the control group, HF/HC diet caused a significant increase in plasma levels of Apelin on all days of the experiment. Treatment of diabetic rats with LC for 14 days had no obvious effect on serum levels of Apelin, while diabetic rats treated with LC for 28 days, showed significantly reduced serum levels of Apelin (P<0.05). LC administration for 14 or 28 days did not change the serum levels of Apelin in healthy rats (Fig .3). 

### L-carnitine influenced *Apelin* and *Apj* expression in cardiac muscle of diabetic rats

**Fig.4:**
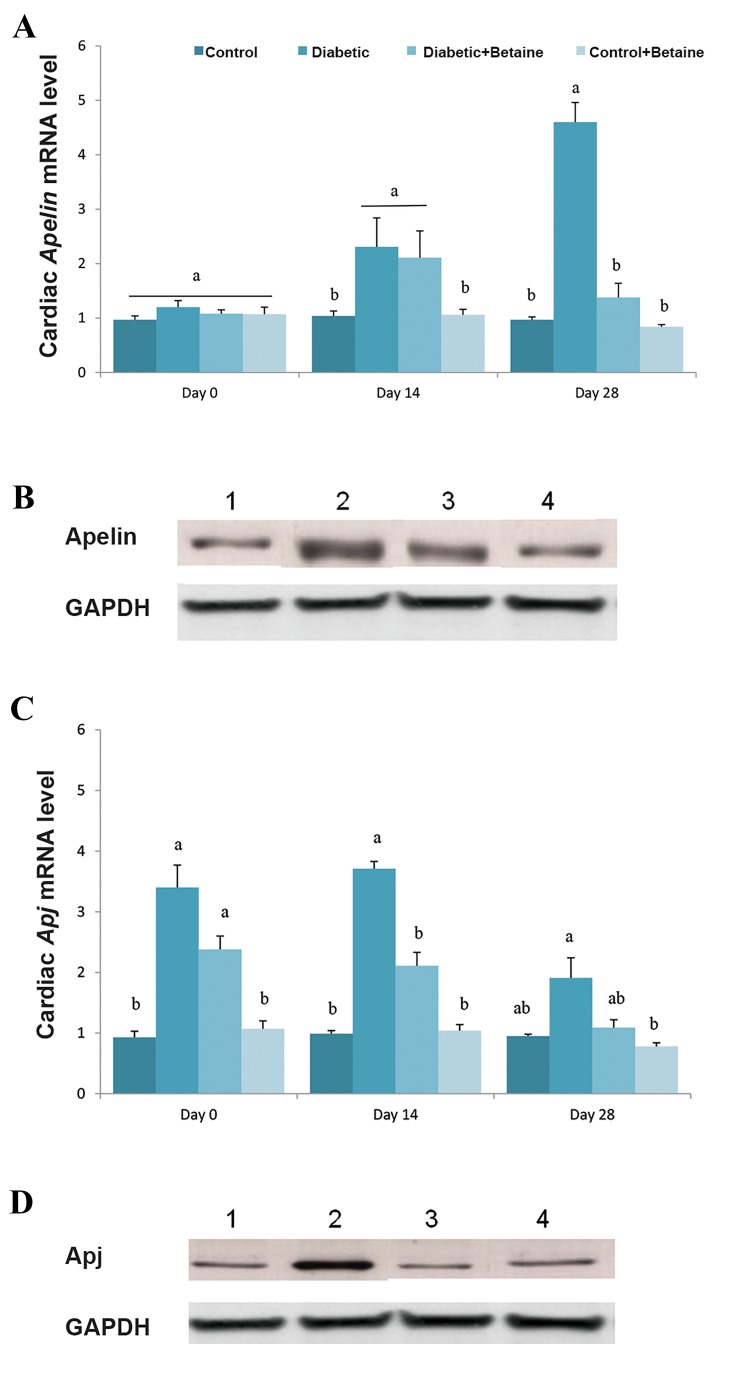
Effect of LC on expression of Apelinand ApjmRNA (on days 0, 14 and 28 of experiment) and protein (on day 28 of experiment) in cardiac muscle of HF/HC diet fed diabetic rats. Rats were fed with regular chow diet (control) or HF/HC diet for 5 weeks (diabetic). HF/HC fed rats were treated with LC (300 mg/kg/day) from the first day of diabetes confirmation for 14 or 28 days (Diabetic+LC, n=5/group). A. ApelinmRNA level, B. Apelin protein level, C. ApjmRNA level, and D. Apj protein level. Data are expressed as means ± SE. Different letters (a, b and c) demonstrate significant differences among groups on each day at P<0.05. LC; L-carnitine and HF/HC; High fat/high carbohydrate.

The expression level of myocardial *Apelin* was significantlyincreased in diabetic rats on days 14 and 28 after diabetesinduction compared to rats that were fed with normal diet(P<0.05, Fig .4A, B). LC treatment for 14 days did not affectthe expression of Apelin in cardiac muscle of diabetic rats, while myocardial *Apelin* expression was down regulated in diabetic animals that received LC for 28 days (P<0.05, Fig .4A, B). 

*Apj* expression was increased in cardiac muscle of diabeticrats 14 days after diabetes induction compared to controlanimals, while after day 14, it reduced to levels similar tothose of control healthy rats. LC treatment significantlyreduced the expression of myocardial *Apj* in diabetic rats (P<0.05, Fig. 4C, D). These results indicated that the LCtreatment efficiently reduced the myocardial over-expressionof Apelin and Apj caused by the HF/HC diet. Treatment ofhealthy rats with LC had no significant effect on myocardial expression of *Apelin* and *Apj* genes (P>0.05, Fig .4A-D). 

### Effect of L-carnitine supplementation on serum lipids

**Fig.5:**
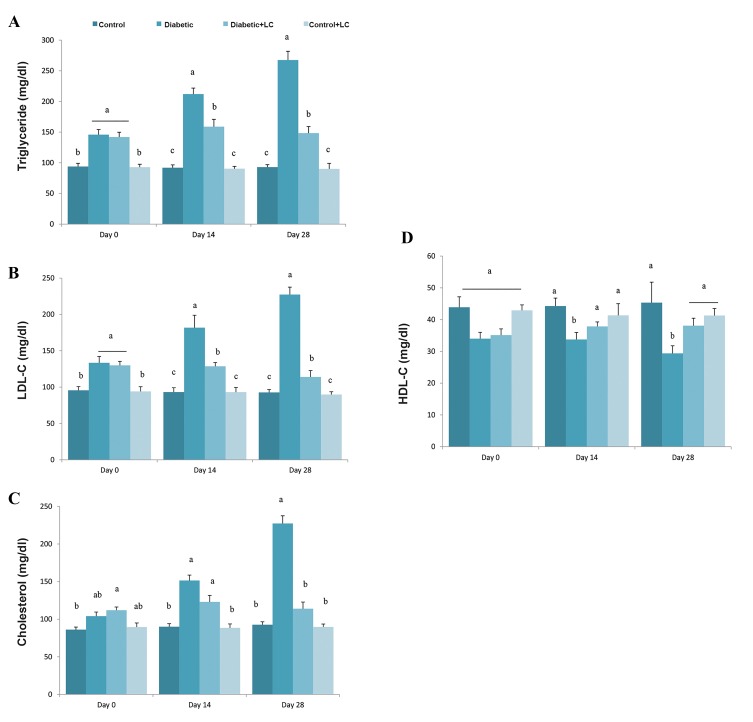
Effect of LC on serum and cardiac muscle levels of TNF-α and IL-1ß of HF/HC diet induced diabetic rats. Rats were fed with regular chow diet (control) or HF/HC diet for 5 weeks (diabetic). HF/HC fed rats were treated with LC (300 mg/kg/day) from the first day of diabetes confirmation for 14 or 28 days (Diabetic+LC, n=5/group). A. Serum TNF-α, B. Cardiac muscle TNF-α, C. Serum IL-1ß, and D. Cardiac muscle IL-1ß. Data are means ± SE. Different letters (a, b and c) demonstrate significant differences between groups in each day at P<0.05. LC; L-carnitine, HF/HC; High fat/high carbohydrate, TNF-α; Tumor necrosis factor-α, and IL-1ß; interleukin-1ß.

Changes in serum lipids including TG, cholesterol, HDL and LDL on days 0, 14 and 21 after treatment of diabetic rats with LC are shown in Figure 5A-D. Significantly higher levels of serum levels of TG and LDL were observed in HF/HC fed group when compared to the regular diet-fed control at the end of HF/HC feeding period (P<0.05). However, cholesterol and HDL levels in diabetic rats were similar to those in control animal. On days 14 and 28 after diabetes induction, serum levels of TG, cholesterol and LDL were elevated, while HDL level were reduced in the HF/HC fed group compared to the control ones (P<0.05, Fig . 5A-D). In diabetic rats that were treated with LC for 14 days, serum levels of TG and LDL were reduced, while those were treated for 28 days showed reduced level of serum TG and LDL, and increased levels of HDL compared to untreated diabetic animals (P<0.05, Fig .5A-D). 

### Alterations of serum tumor necrosis factor-α and interleukin-1ß in diabetic rats treated with L-carnitine

**Fig.6:**
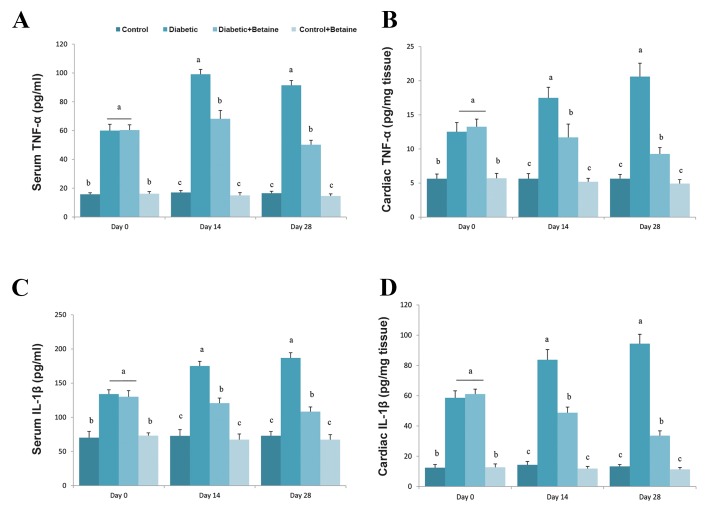
Effect of LC on serum and cardiac muscle levels of TNF-α and IL-1β of HF/HC diet induced diabetic rats. Rats were fed with regular chow diet (control) or HF/HC diet for 5 weeks (diabetic). HF/HC fed rats were treated with LC (300 mg/kg/day) from the first day of diabetes confirmation for 14 or 28 days (Diabetic+LC, n=5/group). A. Serum TNF-α, B. Cardiac muscle TNF-α, C. Serum IL-1β, and D. Cardiac muscle IL-1β. Data are means ± SE. Different letters (a, b and c) demonstrate significant differences between groups in each day at P<0.05. LC; L-carnitine, HF/HC; High fat/high carbohydrate, TNF-α; Tumor necrosis factor-α, and IL-1β; interleukin-1β.

To investigate whether LC could improve inflammation caused by feeding with HF/HC diets, serum levels of TNF-α and IL-1ß were measured in treated and non- treated animals. Serum and tissue levels of TNF-α in all four groups after receiving their respective treatment, are shown in Figure 6A, B. Serum TNF-α level in rats fed with HF/HC diet was higher than that of rats fed with the normal diet on days 0 (2.35 fold), 14 (2.92 fold) and 28 (3.7 fold) (P<0.05, Fig .6A). Cardiac TNF-α level was also higher in diabetic rats compared to control rats on different days after diabetes induction (P<0.05, Fig .6B). LC treatment significantly suppressed serum and tissue levels of TNFa in HF/HC fed rats on days 14 and 28 compared to untreated diabetic animals (P<0.05, Fig .6A, B). LC treatment had no obvious effect on serum and tissue concentrations of TNFa in healthy rats (P>0.05). 

Serum levels of IL-1ß were increased in HF/HC fed rats by 1.85, 2.43 and 2.54 fold on days 0, 14 and 28 after feeding, respectively (P<0.05, Fig .6C). Cardiac IL-1ß level was also increased in a time-dependent manner in diabetic animals compared to healthy rats (P<0.05, Fig .6D). Treatment of diabetic rats with LC for 14 or 28 days significantly reduced the serum and cardiac levels of IL-1ß compared to untreated diabetic rats (P<0.05, Fig .6C, D). 

## Discussion

Obesity is one of the most important causes of CVDs. Obesity can disrupt secretion of adipose-derived adipokines and lead to systemic metabolic dysfunction, inflammation and cardiovascular complications (2-4). According to recent studies, Apelin and its receptor, Apj, have dysregulated expression or secretion patterns in cardiovascular system of obese diabetic rats (28). In the present study, high-fat fed rats with obesity and diabetes were used to investigate the potential effects of LC on Apelin system expression in cardiac muscle.

The results of the present study demonstrated that rats fed with a HF/HC diet showed increased levels of body weight, blood cholesterol, TG, LDL-C and glucose along with hyperinsulinemia and insulin resistance when compared to control animals. In accordance with our results, previous works showed that plasma Apelin level is elevated in patients or animals with type II diabetes and insulin resistance (5, 9, 11, 15, 16). The increased Apelin expression may be due to hyperinsulinemia, since it has been reported that lack of insulin in STZ-treated mice is associated with a decreased expression of Apelin in adipocytes (29).

Our results showed that Apelin and its receptors were upregulated in cardiac muscle of obese diabetic rats. Recently, Alfarano et al. (28) showed that Apelin treatment of obese animals with heart failure accelerates myocardial fatty acid oxidation and improves glucose tolerance. Several studies have provided convincing evidence indicating that mitochondrial dysfunction may be an important event in the development of heart failure in diabetic patients (30). Apelin can attenuate mitochondrial damage in cardiac muscle by increasing mitochondrial DNA content and citrate synthase activity (28). Increased Apelin secretion or expression in diabetic rats, along with hyperinsulinemia, may be a compensatory mechanism to enhance insulin sensitivity and glucose uptake in target tissues such as cardiac muscle. In this regard, recent studies have shown that Apelin stimulates glucose uptake in myotubes, resulting in increased insulin sensitivity and suppression of lipid accumulation in myotubes (31-34).

Our results showed that changes in metabolic indices were associated with increased serum and tissue inflammatory markers including TNFa and IL-1ß. These factors have important roles in cardiovascular dysfunctions in animals and humans with obesity, diabetes and insulin resistance (30, 33). Recent *in vivo* and *in vitro* findings have shown that TNF-α induces *Apelin* gene expression in obese mice. Furthermore, short-term exposure to an intra peritoneal.injection of TNF-α in C57Bl6/J mice increased Apelin expression in adipose tissue and enhanced Apelin plasma levels (33). These results support our hypothesis that inflammatory factors such as TNF-α upregulates the Apelin axis which, in turn, modulates multiple physiological processes and may contribute to Apelinmediated attenuation of cardiac dysfunction. Based on the above findings, it might be concluded that up regulation of Apelin in cardiac muscle of diabetic rats may improve the function of cardiac muscle in this condition and may attenuate the pathophysiological complications in patients with heart failure.

Our results showed that LC, when added to the drinking water, attenuates increased *Apelin* and *Apj* gene expression in cardiac muscle and reduces serum levels of *Apelin* diabetic rats; these changes were associated with reduced insulin resistance indices and serum inflammatory markers. To the best of our knowledge, this is the first report showing cardioprotective effects of LC in diabetes and obesity conditions and its association with modulation of Apelin axis in cardiac muscle. 

LC supplementation may be beneficial in obesity and diabetes conditions as in obese rats with insulin resistance, it was shown that LC supplementation improves glucose tolerance and increases total energy expenditure (35). The molecular mechanism through which LC down regulates *Apelin* and *Apj* expression in cardiac muscle of diabetic rats, is unknown. 

Previous study has shown that weight loss can lead to significant reduction of adipose tissue *Apelin* expression; in the present study, LC treatment led to a weight loss in diabetic rats compared to untreated ones (36). These findings support the possible role of weight reduction on Apelin expression in cardiac muscle. Furthermore, treatment of diabetic rats with LC significantly reduced the serum levels of IL-1ß and TNF-α (37). Because elevated inflammatory cytokines in obesity can accelerate the expression and secretion of *Apelin*, it is hypothesized that down regulation of cardiac *Apelin* in obese diabetic rats treated with LC may be associated with anti-inflammatory action of LC. 

In previous studies, the relationship between cardiomyopathy, cardiac arrhythmia and heart failure due toaccumulation of long-chain fatty acids in the absence of LCor its functional derivatives has been proven (38). In obesepatients died with insulin resistance, severe heart failure andmyocardial infarction have been reported concomitant withvery low levels of serum LC (39). Apelin axis can improvemetabolic and inflammatory disturbances in cardiac muscleof patients with CVDs. Thus, it seems that following LCtreatment, by improving the metabolic and inflammatorycomplications, Apelin expression was reduced, suggestingthat an increase in Apelin axis expression may reflect acompensatory mechanism against development of insulin resistance complications in cardiac muscle of obese patients.

## Conclusion

The results of the current study revealed that cardiac *Apelin* and *Apj* expression were increased in the HF/HC fed rats and these changes were significantly correlated with increased serum levels of Apelin and insulin, body weight, insulin resistance, inflammatory markers and the atherogenic lipid profile. Treatment of diabetic rats with LC resulted in down regulation of Apelin axis in diabetic rats concomitant with improving insulin resistance and inflammatory markers and weight loss. These results suggest that LC acts as novel regulator of Apelin axis in cardiac muscle that can improve cardiac complications in diabetic patients. 
